# Levels of vascular endothelial growth factor and filtration surgery outcomes in diabetic patients with acute primary angle closure

**DOI:** 10.3389/fmed.2026.1867803

**Published:** 2026-07-14

**Authors:** Tianwei Qian, Mingshui Fu, Hao Zhou, Zhihua Zhang, Ying Hu

**Affiliations:** 1 Department of Ophthalmology, Shanghai General Hospital, Shanghai Jiao Tong University, Shanghai, China; 2 National Clinical Research Center for Eye Diseases, Shanghai, China; 3 Shanghai Key Laboratory of Ocular Fundus Diseases, Shanghai, China; 4 Shanghai Engineering Center for Visual Science and Photomedicine, Shanghai, China; 5 Shanghai Engineering Center for Precise Diagnosis and Treatment of Eye Disease, Shanghai, China

**Keywords:** acute primary angle closure, aqueous humor, diabetic patient, filtration surgery, vascular endothelial growth factor

## Abstract

**Purpose:**

This study aimed to compare aqueous humor vascular endothelial growth factor (VEGF) levels in diabetic patients who had experienced acute primary angle closure (APAC) and to evaluate the relationship between VEGF concentrations and outcomes following filtration surgery.

**Methods:**

In this prospective study, aqueous humor specimens were obtained from 46 eyes of diabetic individuals with a history of APAC. VEGF levels were determined. Intraocular pressure (IOP) was assessed using Goldmann applanation tonometry. Anterior segment optical coherence tomography (AS-OCT) was employed to evaluate bleb morphology, and a bleb score was derived based on bleb dimensions and reflectivity. Follow-up examinations were performed at 1 week, and at 1, 3, 6, 12, and 18 months postoperatively.

**Results:**

All eyes underwent combined phacoemulsification, intraocular lens (IOL) implantation, and trabeculectomy, and were followed for 18 months. Based on surgical outcomes, eyes were categorized into a success group (34 eyes) and a failure group (12 eyes). Aqueous VEGF concentrations were markedly elevated in the failure group (*P* = 0.0018). Both univariate and multivariate analyses identified higher aqueous humor VEGF level was associated with surgical failure [univariate: *P* = 0.021, odds ratio (*OR*) = 9.826; multivariate: *P* = 0.030, *OR* = 8.542]. When eyes were stratified according to VEGF level, the low-VEGF group exhibited a significantly higher success rate compared with the high-VEGF group (overall success: *P* = 0.0276; complete success: *P* = 0.0073). Moreover, VEGF levels showed a significant positive correlation with bleb scores (Spearman's ρ =0.6840, 95% *CI*: 0.4845–0.8158, *P* < 0.0001).

**Conclusions:**

In this small cohort of diabetic patients with a history of APAC, higher aqueous humor VEGF level was associated with less favorable filtration surgery outcomes. These findings are exploratory and suggest that VEGF may warrant further investigation as a potential marker. However, external validation in larger prospective cohorts is needed before clinical application.

## Introduction

Acute primary angle closure (APAC) is an ophthalmic emergency typically resulting from the sudden obstruction of the trabecular meshwork within the anterior chamber angle. Its clinical features include a rapid and pronounced rise in intraocular pressure (IOP), conjunctival injection, corneal edema, ocular discomfort, frontal headache, as well as nausea and/or vomiting (1–3). In certain patients, significant peripheral anterior synechiae and persistently elevated IOP may persist even after the resolution of the acute episode. The primary goal of treatment is IOP reduction, which is generally achieved through filtration surgery. Trabeculectomy, a standard glaucoma filtering procedure, aims to lower IOP by establishing an artificial drainage pathway from the anterior chamber to the subconjunctival space, thereby forming a filtering bleb (4). The creation of this bleb helps reduce IOP following trabeculectomy, and its postoperative preservation is critical to the long-term success of the surgery (5, 6).

Vascular endothelial growth factor (VEGF) stimulates the growth of vascular endothelial cells and increases vascular permeability (7). Beyond its role in angiogenesis, VEGF may function as an early trigger of fibrosis and participate in wound healing processes (8–10). Elevated VEGF levels encourage scar formation in cutaneous wounds through collagen deposition, whereas VEGF neutralization reduces both angiogenesis and dermal fibrosis (11). Many studies have shown that the inhibition of VEGF results in reduced scar formation at the trabeculectomy bleb and improves the success of glaucoma surgery (12–15). To date, however, the association between aqueous humor VEGF levels and trabeculectomy results in diabetic patients with APAC has not been thoroughly investigated.

In the present study, we evaluated VEGF levels in the aqueous humor at the time of glaucoma surgery and investigated correlations between VEGF levels and surgical outcomes at 18 months after phacoemulsification and intraocular lens (IOL) implantation combined with trabeculectomy in diabetic patients with APAC.

## Methods

### Patients

This study was conducted in accordance with the Declaration of Helsinki and was approved by the ethics committee of Shanghai General Hospital (No. of ethic committee approval: 2019KY113). All patients were diagnosed with APAC at Shanghai General Hospital, which was also the study center where all subsequent treatments, surgeries, and follow-up examinations were performed. Written Informed consent was obtained from each patient. All patients underwent a thorough ophthalmic examination and agreed to receive phacoemulsification and IOL implantation combined with trabeculectomy to reduce IOP. Participants were recruited prospectively and consecutively at Shanghai General Hospital from July 2019 to June 2020. The key inclusion criteria included the following: (1) patients with prior APAC who had experienced an APAC attack within 1 month before admission; (2) patients had uncontrolled IOP under standardized antiglaucomatous medications after laser peripheral iridotomy (LPI); (3) had a clear history of diabetes and good blood glucose control for at least 3 month (HbA1c ranged from 4% to 6%); (4) patients had functionally significant cataract and were willing to undergo phacoemulsification with IOL; (5) moderate non-proliferative diabetic retinopathy (NPDR) or better according the grade of diabetic retinopathy (DR) (16); and (6) agreement to finish 18 months of follow-up. The exclusion criteria were (1) ocular diseases other than APAC; (2) uncontrolled diabetes mellitus (defined as fasting blood glucose ≥7 mmol/L or 2-h postprandial blood glucose ≥11.1 mmol/L); (3) uncontrolled hypertension; (4) eyes with history of intraocular injection of anti-VEGF agents and/or steroids; (5) active ocular inflammation or infection; (6) any history of previous ocular surgery; and (7) an IOP value of ≤ 21 mmHg at all three visits prior to surgery. Some or all of the following medications were used according to patients' IOP and physical status: (1) topical pilocarpine; (2) topical α2-adrenergic agonists; (3) topical β-adrenergic antagonists twice daily, (4) topical steroids four times daily, and (5) topical and/or oral carbonic anhydrase inhibitors. None preoperative steroids were used after eyes were included in the study. Among patients in whom both eyes were eligible, only the first eye undergoing surgical treatment was included in the study.

### Surgical technique and postoperative care

For all the prior APAC patients, phacoemulsification and IOL implantation combined with trabeculectomy was performed by one experienced surgeon (M. Fu). Patients first underwent phacoemulsification and IOL implantation under topical gel anesthesia, and then and the main steps of filtration surgery were similar to those described in our previous study (17, 18). A fornix-based conjunctival flap was created from 11 to 1 o'clock, followed by dissection of a limbus-based 4 × 4-mm scleral flap. Then a sponge soaked with 5-fluorouracil (5-FU, 25 mg/ml) was placed underneath the conjunctival flap for 3 min, followed by irrigation of the surgical area with balanced salt solution. Subsequently, a sclerostomy and basal iridectomy were performed. After previous procedure, the scleral flap was sutured with a single, interrupted 10-0 nylon suture at the two corners and adjustable sutures at the center of the two sides to allow minimal leakage during anterior chamber reconstruction. Finally, the conjunctiva was closed using a 10-0 nylon suture.

A topical antibiotic and topical non-steroidal anti-inflammatory medication were given four times daily for 1 week. Topical corticosteroids were used four times daily for the first week and then tapered over the next months. No additional antimetabolites were used during the whole follow-up. Based on two previous studies (19, 20), postoperative interventions were performed according to bleb formation. First, we performed ocular massage; in cases where the postoperative IOP did not reach target despite this treatment, we loosened the adjustable sutures with forceps. If the bleb became encapsulated and associated with a rise in IOP over 21 mmHg, we performed bleb needling and added IOP-lowering medications. In cases of inadequate IOP control despite these above-mentioned measures, additional surgical procedures were performed as required.

### Collection of aqueous humor

Generally, aqueous humor samples (at least 50–100 μl) were collected at the beginning of phacoemulsification and IOL implantation. All collections were carefully performed without touching any intraocular tissues. Aqueous humor samples were immediately frozen in liquid nitrogen and transferred into a −80 C environment until analyzed.

### Measurement of VEGF levels in aqueous humor

VEGF levels were detected using an assay (Luminex Screening Human Magnetic Assay; R&D Systems, Inc., Minneapolis, MN, USA) and the assay was performed according to the manufacturer's instructions. Fluorescence intensity was acquired and analyzed using software (Luminex xponet3.1; Luminex, Austin, TX, USA).

### Outcome measures

Preoperative IOP and all topical anti-glaucoma medications used in this study were recorded before surgery. Patients were followed up 1 week and 1, 3, 6, 12 and 18 months postoperatively. At each postoperative visit, we measured IOP levels using Goldmann applanation tonometry. The number of anti-glaucoma medications used and any additional surgeries performed were also recorded. We divided patients into two groups, success and failure, based on surgical results. The success group was subdivided into complete success and qualified success (18). Failure was defined as IOP > 21 mmHg or a < 20% reduction from baseline at two consecutive follow-up visits after 3 months, an IOP of 5 mmHg or less at two consecutive follow-up visits after 3 months, reoperation for glaucoma, or loss of light perception vision. Complete success was defined if the above failure criteria were not met, and no supplemental medications were required. Qualified success was defined as not satisfying the above-mentioned failure criteria, but supplemental medications were required (2, 21). The primary endpoint of this study was surgical outcome (success vs. failure) at 18 months postoperatively. We defined reoperation for glaucoma as additional glaucoma surgery requiring a return to the operating room, such as tube shunt placement. We also regarded cyclodestruction as a reoperation for glaucoma, whether performed in the clinic or operating room. Ocular massage, loosening of adjustable sutures, and bleb needling were performed using a slit lamp and were not considered additional glaucoma surgery (22–24). To define surgical failure, IOP values obtained 3 months or later after trabeculectomy were used to avoid the effect of short-term postoperative IOP fluctuations. For the time-to-event analysis, the event time was defined as the time (in months) from surgery to the first of two consecutive postoperative visits (at 3 months or later) at which the failure criteria were met. Eyes that did not meet failure criteria by the end of the 18-month follow-up were censored at the last visit.

Anterior segment optical coherence tomography (AS-OCT) images of the bleb were taken at postoperative 18 month with an imaging system (CASIA SS-1000; Tomey, Nagoya, Japan). An 8^*^8-mm scan of each bleb, including the bleb apex, was acquired. Scoring was carried out according to the novel grading system created by Wen et al. (25). A scale of 0 to 3 using 0.5 increments was applied to the image through the apex of the bleb by comparing it to the reference photographs (25). Larger blebs with greater bleb height and width and with lower reflectivity were given lower grades. When the size and the reflectivity of blebs were not parallel, the thickness and reflectivity of the bleb wall was evaluated. Encapsulated blebs tend to have a scarred wall with high reflectivity. Thus, a hypo reflective thick bleb wall with multiple parallel layers or cyst, suggesting the bleb was functional (26, 27), was given lower grade. The score of a bleb was the mean value of scores given by two experienced graders. An additional grader was assigned to reevaluate the images and gave the final score if there was too much discrepancy (≥1) between the two separated graders.

### Statistical analysis

Commercially available SPSS software (version 22.0; SPSS, Inc., Chicago, IL, USA) and GraphPad Prism (version 8.01, GraphPad software Inc., San Diego, CA, USA) were used for the analyses. Categorical variables (e.g., gender) were expressed as numbers and frequencies, and the differences with regard to gender were assessed using Fisher's exact test. The Kolmogorov–Smirnov test was used to determine whether continuous variables fit with a normal distribution (28). Continuous variables were expressed as the mean ± standard deviation (normally distributed data) or median and interquartile range (abnormal distributed data). The unpaired *t*-test was used for normally distributed data and the Mann–Whitney *U*-test for abnormal distributed data. Univariate and multivariate logistic regression analyses were used to assess variables associated with surgical failure. Due to the limited number of failure events, we performed a reduced multivariate model with fewer covariates to assess the robustness of the VEGF association. This reduced model was chosen on clinical grounds based on clinical importance and was limited to four covariates due to the small number of failure event. The primary statistical analysis was multivariate logistic regression evaluating VEGF as a continuous predictor of the primary endpoint. For logistic regression analyses, VEGF levels were analyzed as a continuous variable. The odds ratio (OR) reported represents the change in odds of surgical failure for each 100 pg/ml increase in aqueous VEGF concentration, as this increment corresponds to a clinically meaningful difference in our cohort. The Wald test was used to calculate *P*-values, and 95% confidence intervals (CIs) were derived using the Wald method. If VEGF was detected to associated with trabeculectomy failure, we divided the patients into two groups according to the level of this cytokine, and Kaplan-Meier survival curves were used to display the success rates of end points over time between groups. Spearman correlation analysis and linear regressions were used to assess the relationship between the VEGF levels and bleb scores assessed by AS-OCT. The coefficient of determination *R*^2^ is used to express the proportion of the variation in the dependent variable explained by the regression model.

## Results

### Patients and surgical results

In total, 46 previous APAC eyes of diabetic patients were included and these participants completed the 18-month follow- up period. We classified 34 eyes (73.9%) into the success group (27 eyes belonged complete success and 7 eyes belonged qualified success), and 12 eyes (26.1%) into the failure group during the follow-up period. The clinical characteristics of both groups are shown in Table 1. There were no significant differences between both groups considering gender, age, preoperative IOP, numbers of antiglaucoma eyedrops, HbA1c, the NPDR grade, and the interval between the onset of APAC and surgery. However, the VEGF levels were significantly higher in the 12 eyes in the failure group compared to the 34 eyes in success group (*P* = 0.0018; Table 1).

**Table 1:** Characteristics of 46 previous APAC patients included.

Characteristics	All previous APAC eyes	Success group	Failure group	*P*
Patients (eyes), No.	46 (46)	34 (34)	12 (12)	–
Male/female	21/25	16/18	5/7	0.7472
Age, *y, M* ±*SD*	67.4 ± 7.8	67.7 ± 8.5	66.6 ± 5.9	0.6826
Preoperative IOP, mmHg, *M* ±*SD*	34.0 ± 5.8	33.5 ± 6.5	35.4 ± 3.3	0.3418
HbA1c, %, *M* ±*SD*	5.15 ± 0.60	5.19 ± 0.62	5.03 ± 0.56	0.4593
**NPDR grade**	
Non-DR	14	11	3	0.8918
Mild NPDR	18	13	5	
Moderate NPDR	14	10	4	
Number of antiglaucoma eyedrops, *M* ±*SD*	2.2 ± 0.8	2.2 ± 0.9	2.3 ± 0.8	0.9595
Interval between the onset of APAC and trabeculectomy, d, *M* ±*SD*	12.5 ± 4.9	12.3 ± 5.1	13.1 ± 4.4	0.6237
VEGF, pg/ml, median (IQR)	426.4 (516.3)	364.5 (435.2)	820.4 (354.1)	***0.0018***

APAC, acute primary angle closure; IOP, intraocular pressure; VEGF, vascular endothelial growth factor; DR, diabetic retinopathy; NPDR, non-proliferative diabetic retinopathy; M, mean; SD, standard deviation; IQR, interquartile range. Bold italic values indicate statistically significant results.

Table 2 shows the postsurgical IOP-lowering interventions in the success and failure group. There was no significant difference in ocular massage or suture lysis (*P* = 0.5940 and *P* = 0.4124, respectively) between the groups. However, four eyes (11.8%) in the success group and five eyes (41.7%) in the failure group required needling revision, and this difference was significant (*P* = 0.0388).

**Table 2:** Postoperative interventions and complications in the success and failure group.

Interventions/Complications	Success group, *n* (%)	Failure group, *n* (%)	*P*
**Postoperative interventions**			
Ocular massage	3 (8.8%)	2 (16.7%)	0.5940
Suture lysis	5 (14.7%)	3 (25.0%)	0.4124
Needling revision	4 (11.8%)	5 (41.7%)	***0.0388***
**Complications**			
Corneal epitheliopathy	2 (5.9%)	1 (8.3%)	>0.9999
Hyphema	1 (2.9%)	1 (8.3%)	0.4580
Iridocyclitis	2 (5.9%)	2 (16.7%)	0.2758
Malignant glaucoma	0 (0%)	1 (8.3%)	0.2609
Shallow anterior chamber	1 (2.9%)	0 (0%)	>0.9999
Transconjunctival oozing	0 (0%)	0 (0%)	–
Endophthalmitis	0 (0%)	0 (0%)	–

Bold italic values indicate significant results.

Surgical complications in both groups are also shown in Table 2. There was no significant difference between the two groups regarding all complications (all *P* > 0.05), with six adverse events in the success group and five adverse events in the failure group. All adverse events recorded were either managed medically or recovered without treatment. No severe complications, such as endophthalmitis, were found during follow-up in the two groups.

### Correlation between VEGF levels and surgical results

We performed univariate and subsequent multivariate analyses to further assess the association between potential variables and surgery results. Univariate analysis revealed that the AH level of VEGF was significantly associated with filtration surgery outcome (*P* = 0.021, *OR* = 9.826); however, age, gender, preoperative IOP, HbA1c, the number of antiglaucoma eyedrops, interval between the onset of APAC and NPDR grade were not (Table 3).

**Table 3:** Results of univariate analysis of surgery outcomes.

Variable	*OR*	95% *CI*	*P* (Wald)
Age	0.982	0.902–1.069	0.675
Gender	1.244	0.329–4.708	0.747
Preoperative IOP	1.057	0.944–1.185	0.335
HbA1c	1.534	0.505–4.651	0.451
Number of antiglaucoma eyedrops	1.021	0.465–2.241	0.958
Interval between the onset of APAC and surgery	1.035	0.904–1.186	0.615
NPDR grade	1.204	0.516–2.811	0.667
VEGF	9.826	1.382–69.863	***0.021***

VEGF was analyzed as a continuous variable (pg/ml); OR is reported per 100 pg/ml increment. Bold italic values indicate significant results.

Considering the small sample size, we performed a reduced multivariate model including only four variables based on clinical importance: VEGF level, age, HbA1c and preoperative IOP. The multivariate analysis also revealed that the only VEGF level remained significantly associated with surgical outcome (*P* = 0.030, *OR* = 8.542; Table 4).

**Table 4:** Results of multivariate analysis of surgery outcomes.

Variable	*OR*	95% *CI*	*P* (Wald)
Age	0.933	0.837–1.040	0.212
Preoperative IOP	1.142	0.962–1.356	0.128
HbA1c	2.770	0.636–12.048	0.175
VEGF	8.542	1.213–60.153	***0.030***

VEGF was analyzed as a continuous variable (pg/ml); OR is reported per 100 pg/ml increment. Bold italic values indicate significant results.

**Figure 1:**
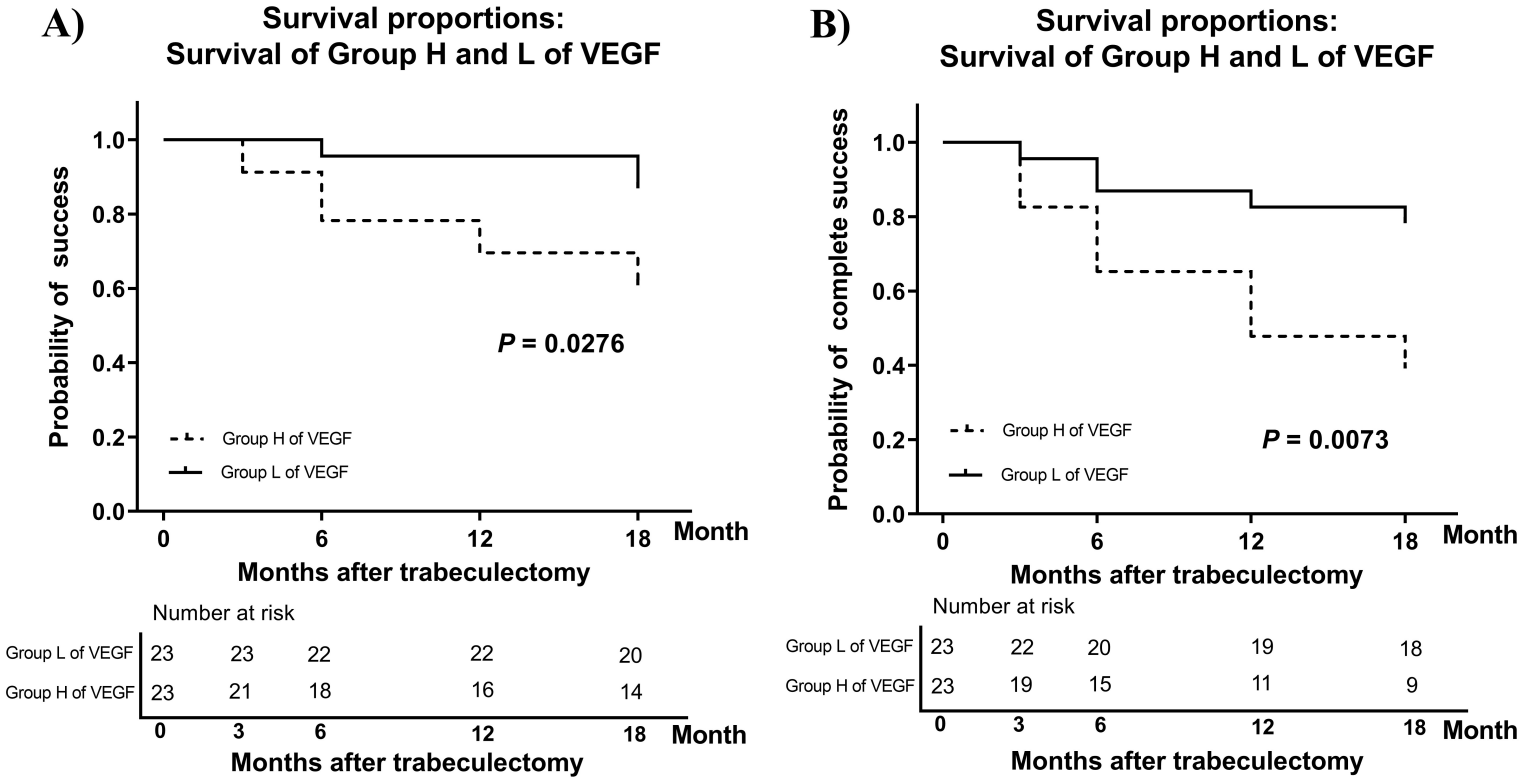
Kaplan-Meier survival plot of two groups according to aqueous humor VEGF level: eyes with VEGF value < 426.4 pg/ml (group L, 23 eyes, solid line) and eyes with VEGF value ≥426.4 pg/ml (group H, 23 eyes, dashed line). **(A)** The probability of success of surgery for eyes with low VEGF levels was significantly higher than that for eyes with high VEGF levels (*P* = 0.0276). **(B)** The probability of complete success for eyes with low VEGF levels was also significantly higher than that for eyes with high VEGF levels (*P* = 0.0073).

We subsequently divided the 46 previous APAC patients into two groups based on the level of VEGF. Twenty-three eyes with VEGF levels lower than the median value (< 426.4 pg/ml) were included in group L, and other 23 patients with VEGF level higher than or equal to the median value (≥426.4 pg/ml) were included in group H. The postoperative outcomes including postoperative IOP, anti-glaucomatous eye drop usage, and success rates are shown in Table 5. The success rates at 18 months after surgery were 87.0% and 60.9% in group L and group H, respectively. The complete success rates at 18 months were 78.3% and 39.1% in group L and group H, respectively. The results revealed that the differences in surgery outcomes at 18 months between both groups were significant (*P* = 0.0439 for overall success, and *P* = 0.0070 for complete success). The detailed risk, event counts, and survival estimates were provided in Supplementary Tables S1, S2. The Kaplan–Meier survival plots were drawn to investigate the success rate between both groups (Figure 1). Eyes with low VEGF levels (group L) had significantly higher overall success rate and complete success rate over the 18 months of follow-up compared to eyes with high VEGF levels (group H; *P* = 0.0276, Figure 1A; and *P* = 0.0073, Figure 1B, respectively).

**Table 5:** Surgical results between groups with low and high levels of VEGF.

Characteristics	Previous APAC with low levels of VEGF, group L	Previous APAC with high levels of VEGF, group H	*P*
Patients, No.	23	23	–
VEGF level, mean, pg/ml	275.54	762.38	***<0.0001***
**Probability of success, %**			
3 months	100	91.3	0.1482
6 months	95.7	78.3	0.0799
12 months	95.7	69.6	***0.0196***
18 months	87.0	60.9	***0.0439***
**Probability of complete success, %**			
3 months	95.7	82.6	0.1553
6 months	87.0	65.2	0.0839
12 months	82.6	47.8	***0.0133***
18 months	78.3	39.1	***0.0070***
IOP at 18 months postoperatively	14.2 ± 2.6	15.8 ± 3.8	0.1026
Number of glaucoma eyedrops at 18 months postoperatively	0.1 ± 0.4 (0–1)	0.4 ± 0.7 (0–2)	0.1102

APAC, acute primary angle closure; IOP, intraocular pressure; VEGF, vascular endothelial growth factor; bold italic values indicate statistically significant results.

### Correlation between VEGF level and bleb score

**Figure 2:**
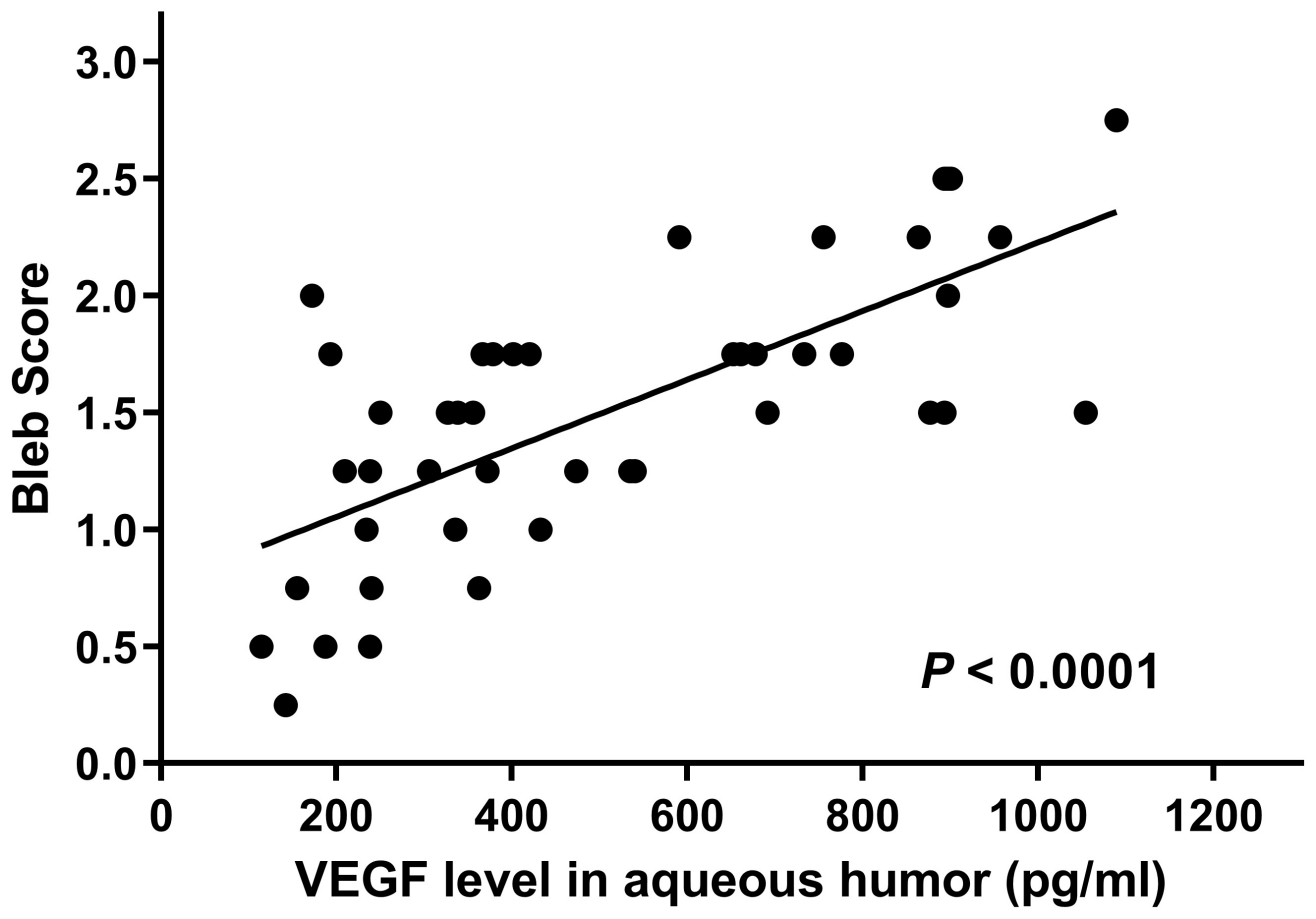
Scatter plot showing the correlation between aqueous humor VEGF levels and AS-OCT bleb scores at postoperative 18 months. Spearman's correlation coefficient (ρ = 0.6840, 95% *CI*: 0.4845 to 0.8158, *P* < 0.0001) indicates a significant positive correlation. The linear regression line (*P* < 0.0001, *R*^2^ = 0.5064) is shown for descriptive purposes as a sensitivity analysis.

To quantify the agreement between the two primary graders, we have now calculated the intraclass correlation coefficient (ICC) using a two-way random effects model for absolute agreement. The ICC for the 46 bleb scores was 0.89 (95% *CI*: 0.82–0.94), indicating excellent reliability. For the primary Spearman correlation, we reported the correlation coefficient was 0.6840 (95% *CI*: 0.4845 to 0.8158, *P* < 0.0001). Also, we have retained the linear regression. The relationship between level of VEGF with bleb score at postoperative 18 months was evaluated (Figure 2). The VEGF level had significantly positive correlation with bleb score (*P* < 0.0001, *R*^2^ = 0.5064), and the slope was 0.001466 (95% *CI*: 0.001027 to 0.001906) per pg/ml increase in VEGF. The bleb score (*y*) was related to the VEGF level (*x*) according to the formula *y* = 0.7607+ 0.001466*x*.

## Discussion

Phacoemulsification and IOL implantation combined with trabeculectomy is currently the prevailing operative technique for previous APAC. An unobstructed artificial drainage route from the anterior chamber to the subconjunctival space is the key to success. Poor outcomes are usually due to the excessive subconjunctival scarring at the filtering bleb and inhibition of scarring after trabeculectomy has been attracting increasing attention in recent years.

It is well-known that VEGF is a potent angiogenesis cytokine. It can be expressed and produced by various cell types that line the anterior and posterior segments, which includes the corneal endothelium, iris pigment epithelium, retinal pigment epithelium, retinal ganglion cells, astrocytes, and its level is elevated significantly during ischemia and hypoxia (29, 30). It has been reported that VEGF was significantly increased in aqueous humor of APAC eyes (30, 31). Our previous study enrolled patients without history of diabetes (18), and the results showed that the VEGF level in failure group were higher compared to the success group. However, there was no significant difference. Studies have revealed increased level of VEGF in the eyes of diabetic patients (32). We postulated that the higher level of VEGF in aqueous humor might act as an initial activator of fibrosis and was significantly associated with surgical results in diabetic patients.

In the present study, aqueous humor samples collected from 46 previous APAC eyes in diabetic patients were analyzed. Our results showed that the VEGF level was significantly higher in failure group than in success group. Thus, it is reasonable to hypothesize that increased VEGF levels in the aqueous humor entered filtration blebs through the pathway created by the trabeculectomy, resulting in the enhanced formation of fibrosis tissue. Considering AS-OCT is a very convenient way to observe wound healing in filtering blebs, and AS-OCT is used to assess bleb size and internal reflectivity at 18 months postoperative in relation to VEGF levels. Our results demonstrated that higher VEGF levels in the aqueous humor were associated with increasing AS-OCT bleb scores (smaller bleb size and higher scarring). This reaffirmed that high VEGF levels might lead to surgical failure.

Ranibizumab (Lucentis, Genentech, San Francisco, CA, USA) and Bevacizumab (Avastin, Genentech, San Francisco, CA) are two commonly used anti-VEGF agents, which were widely used in clinical and experimental animals. Memarzadeh et al. (33) reported subconjunctival injections of 1.25 mg of bevacizumab could significantly improve bleb survival in rabbit eyes compared with 5 mg of 5-FU or 0.1 ml of balanced salt solution. In a pilot study, a trend for increased central bleb avascularity was observed after injection of Bevacizumab (34). In another pilot study, Kahook found compared to use of topical Mitomycin-C alone that combination intravitreal ranibizumab and topical Mitomycin-C at time of trabeculectomy resulted in more diffuse blebs with less vascularity (35). Freiberg et al. (36) also reported postoperative subconjunctival bevacizumab injection could reduce scarring after trabeculectomy. Furthermore, long term elevated intraocular pressure can cause optic nerve damage. Song et al. (37) found Ranibizumab attenuated optic nerve injury by reducing of VEGF in aqueous humor of glaucoma rat model.

The mechanism of the effect of VEGF on scarring has been widely concerned. Park et al.'s (38) findings suggest that VEGF has potential effects on the TGF-β1/Smad/Snail pathway involved in myofibroblast transformation. This gives an experimental basis for the use of anti-VEGF agents in glaucoma surgery. Besides that, there is an early “acute inflammatory” condition in current APAC patients, which is consistent with clinical findings such as ocular pain, eye redness, and excessive increases in IOP. And Cheng et al. (39) found that found that Bevacizumab could significantly improve the outcome of filtration surgery by inhibiting angiogenesis factor, VEGF, and inflammation factors, TGF-β1 and TGF-β2 dramatically in rat filtration surgery model. Therefore, VEGF, one of the most important inducers of angiogenesis and vascular permeability, also has a strong link with inflammation and immunity. Different VEGF isoforms play differential roles in scar formation. VEGF_165_ and VEGF_121_ predominantly affect blood vessel growth, whereas VEGF_189_ may be more important in fibrosis (14).

Several diabetes-related factors that may influence VEGF expression and wound healing were not captured in this study. First, diabetes duration has been shown to correlate with cumulative microvascular damage and baseline VEGF levels, but we did not have reliable duration data for all patients. Second, systemic vascular complications (e.g., diabetic nephropathy, macrovascular disease) could reflect a more pro-inflammatory and pro-angiogenic systemic milieu, potentially affecting filtering bleb fibrosis. Third, glycemic variability has emerged as a key determinant of diabetic complications and may affect postoperative healing trajectories. Our cohort was limited to patients with good glycemic control and non-proliferative diabetic retinopathy or better, which partially reduces but does not eliminate confounding by these factors. Future prospective studies should systematically collect data on diabetes duration, systemic diabetic complications, and continuous glucose monitoring-derived glycemic variability metrics to more comprehensively adjust for these confounders.

It is important to acknowledge that the dichotomisation of VEGF levels into low- and high-VEGF groups using the median value (426.4 pg/ml) was performed for exploratory purposes only. While this approach facilitated the generation of Kaplan–Meier survival curves for visual illustration of surgical outcomes over time, it has recognized methodological limitations. Median splitting of a continuous biomarker reduces statistical power, discards individual-level information, and may introduce an artificial threshold that lacks biological or clinical justification. Our primary analyses, as shown in Tables 3, 4, higher aqueous humor VEGF level was associated with surgical failure. The exploratory median split was not intended to establish a clinically definitive cutoff, nor was it validated in an independent cohort. Future studies employing receiver operating characteristic (ROC) analysis and external validation are warranted to determine whether a clinically meaningful VEGF threshold exists for predicting filtration surgery outcomes in diabetic patients with prior acute primary angle closure. Given the limited number of failure events (*n* = 12), our multivariable model included only four covariates to avoid overfitting. The resulting estimates, particularly the wide confidence intervals for VEGF, should be interpreted with caution. Future studies with larger sample sizes and more failure events are needed to assess whether VEGF is associated with surgical outcomes in this population.

Several limitations apply to this study. First, although this study was prospectively designed, the sample size was relatively small. However, this is a balanced study with respect to normal variable such as age and sex, and variables more difficult to control such as pre-operative IOP. Second, the activity and level of VEGF may vary during the course of the disease. This makes it important to examine VEGF in various tissue regions and at different stages of the disease. Future animal studies are needed to verify our results by dynamic observation. Nevertheless, our results show that VEGF levels are correlated with surgical results for diabetic patients, providing room for further research aimed at targeting valuable therapeutic factors.

In conclusion, in this exploratory study of diabetic patients with prior APAC, aqueous humor VEGF levels were associated with surgical outcomes. Higher VEGF levels correlated with a higher likelihood of trabeculectomy failure in this small cohort. These results are hypothesis-generating and suggest that VEGF may be a promising target for future research aimed at reducing subconjunctival fibrosis after glaucoma surgery. However, given the limited sample size and lack of external validation, these findings should be interpreted with caution. Larger, multicentre prospective studies are needed to confirm whether VEGF serves as an independent predictive biomarker and to determine whether anti-VEGF interventions improve filtration surgery outcomes in this population.

## Data Availability

The raw data supporting the conclusions of this article will be made available by the authors, without undue reservation.
