# Supplementary material for: Levels of vascular endothelial growth factor and filtration surgery outcomes in diabetic patients with acute primary angle closure
Source: Front Med (Lausanne). 2026 Jul 14;13:1867803. doi: 10.3389/fmed.2026.1867803 (PMC13407501; doi:10.3389/fmed.2026.1867803)
Supplement: Supplementary file 2 [file Table_2.docx]

**Table S2. Numbers at risk, event counts and survival estimate in Low and High levels of VEGF comparing complete success rate at different time points**

| Time point (months) | VEGF group | Number at risk | Cumulative events | Survival estimate (%) |
| --- | --- | --- | --- | --- |
| 0 | Low | 23 | 0 | 100 |
| 0 | High | 23 | 0 | 100 |
| 3 | Low | 22 | 1 | 95.7 |
| 3 | High | 19 | 4 | 82.6 |
| 6 | Low | 20 | 3 | 87.0 |
| 6 | High | 15 | 8 | 65.2 |
| 12 | Low | 19 | 4 | 78.3 |
| 12 | High | 11 | 12 | 47.8 |
| 18 | Low | 18 | 5 | 78.3 |
| 18 | High | 9 | 14 | 39.1 |
